# A fellows first night on call: managing the chaos in the storm

**DOI:** 10.1186/s12909-026-09623-8

**Published:** 2026-06-30

**Authors:** Daniel B. Hanna, Douglas W. Harrington, Sankalp Patel, Mazen Albaghdadi, Michael Flynn, Luis H. Paz Rios, Robert J. Cubeddu, Viviana Navas

**Affiliations:** 1 NCH Rooney Heart Institute, 350 7th Street North, Naples, FL 34102 USA; 2 Department of Simulation, Naples Community Hospital Healthcare System, Naples, USA

**Keywords:** Cardiology fellowship, Simulation-based education, Night-float orientation, High-fidelity simulation, Clinical decision-making

## Abstract

**Introduction:**

Traditional cardiology orientation training offers limited exposure to acute overnight decision-making under high cognitive load. Simulation provides a safe, reproducible environment for deliberate practice and reflection.

**Methods:**

A multi-scenario, high-fidelity “night-float” simulation was implemented for incoming cardiology fellows at the NCH Rooney Heart Institute. Fellows managed sequential acute cardiovascular emergencies with embedded nurses and standardized patients, followed by structured debriefing using the PEARLS framework. Pre- and post-simulation surveys assessed confidence across clinical, procedural, and communication domains.

**Results:**

Five fellows participated. Post-simulation confidence improved across all domains, with the greatest gains in decision-making and leadership under pressure. Faculty observed enhanced situational awareness and prioritization as scenarios progressed.

**Conclusions:**

This cardiology night-float simulation is a feasible, reproducible high-impact orientation model. Early performance observations enabled individualized feedback and follow-up, supporting mastery learning and readiness for overnight call responsibilities.

**Supplementary Information:**

The online version contains supplementary material available at https://doi.org/10.1186/s12909-026-09623-8.

## Introduction

Traditional cardiology fellowship on-boarding offers limited structured simulation education in regards to acute overnight decision-making, which often occurs under conditions of high cognitive load and time pressure [1]. Early trainees entering their first clinical rotations, especially night coverage/call, may face multiple simultaneous emergencies without the benefit of structured rehearsal, contributing to anxiety, delayed prioritization, and communication breakdowns. Simulation-based learning has emerged as a powerful tool to bridge this gap, allowing learners to practice high-stakes scenarios within a psychologically safe, controlled environment that promotes reflection, feedback, and deliberate practice [2–4]. 

While simulation has been integrated into emergency medicine and critical care education [5, 6] its application within cardiology training, particularly for replicating the complexity of overnight triage and decision-making, remains limited [7]. More recent studies (2022–2025) have begun to expand simulation into cardiovascular fellowship curricula (including TEE, interventional procedures, and team decision-making) and support its effectiveness in knowledge, technical skills, and confidence among cardiology trainees [8–11]. Existing cardiology-focused simulations often target discrete procedural or diagnostic skills (e.g., transvenous pacing, echocardiography, or cardiac arrest management) rather than the dynamic interplay of multiple overlapping urgencies requiring prioritization, interprofessional coordination, and leadership [12, 13].

To address this gap, we developed and piloted a multilayered, sequential simulation for incoming first-year cardiology fellows as part of fellowship orientation. The curriculum aimed to replicate the realistic experience of a “night-float” call—requiring rapid triage of concurrent cardiovascular emergencies and facilitating the transition from residency-level decision making to fellowship level decision making. We hypothesized that participation in this simulation would enhance fellows’ confidence, communication, and clinical decision-making across diverse acute cardiology scenarios.

## Methods

This educational innovation was conducted at the Judith and Marvin Herb Family Simulation Center at the Naples Comprehensive Health (NCH) and the Rooney Heart Institute as part of the 2025 cardiology fellowship orientation program. Five incoming first-year cardiology fellows each completed the simulation individually. Each fellow completed all five simulation scenarios consecutively, followed by a single structured debriefing at the conclusion of the session. Scenarios were run sequentially, and fellows did not observe other participants’ sessions to preserve psychological safety and authenticity. The simulation was facilitated by cardiology attendings, simulation medical director, and embedded bedside nurses to ensure clinical fidelity.

### Simulation design

The “Cardiology Night-Float Simulation” was developed and organized by the lead author in collaboration with the NCH Simulation Center staff, fellowship leadership, and core cardiology faculty. The simulation utilized high-fidelity adult mannequins capable of displaying dynamic vital signs, heart sounds, palpable pulses, ECG rhythm changes, and real-time physiologic responses to interventions such as cardioversion. One simulation included a standardized patient for realism in communication, consent, and family interaction scenarios. Specialized task trainers were incorporated where appropriate, including point-of-care ultrasound, synchronized cardioversion and a transvenous pacing (TVP) system used to simulate pacemaker malfunction and post–transcatheter aortic valve replacement (TAVR) bradycardia management. The simulation design intentionally mirrored the escalating cognitive and procedural demands of an overnight call, challenging fellows to prioritize and manage multiple time-sensitive cardiovascular emergencies under uncertainty.

Each participant progressed through five interconnected scenarios, each representing a distinct domain of acute inpatient cardiology (Table 1):

**Table 1:** Overview of the Five Night-Float Simulation Scenarios With Associated Clinical Problems, Learning Objectives, and Workflow Requirements

Simulation Scenario Summary Table			
Scenario	Primary Diagnosis	Key Learning Objectives	Workflow Requirements
1. Post-TAVR* Bradycardia / Loss of Capture	Intermittent high-grade AV block; loss of capture after TAVR.	• Recognize symptomatic bradycardia. • Troubleshoot pacing. • Communicate effectively with appropriate consultants. • Decide when emergent backup pacing is needed.	• PACERMAN™ • Pre- and post-procedure EKG’s*
2. Suspected Cardiac Tamponade After CABG*	Post-operative pericardial effusion; concern for tamponade.	• Differentiate effusion vs. tamponade using POCUS*. • Order formal TTE. • Communicate and deescalates findings and rationale for conservative management. • Notify CTS/ICU* appropriately.	• Intensivist • Echocardiogram Demonstrating pericardial effusion without tamponade physiology
3. Atrial Fibrillation with RVR*	AF with RVR causing hemodynamic instability.	• Rapid diagnostic evaluation. • Select appropriate pharmacologic therapy. • Perform synchronized cardioversion. • Ensure anticoagulation post-DCCV*.	• ZOLL monitor with defibrillator Pads
4. Chest Pain with Troponin Rise → STEMI*	ACS with rising troponin; possible STEMI.	• Prompt STEMI recognition. • Activate catheterization lab. • Start guideline-directed ACS therapy. • Communicate with patient, family, and IC team.	• ECG showing acute MI • Standardized Patient • Interventional cardiologist
5. Acute Decompensated HFrEF*	HFrEF exacerbation, possible cardiogenic shock.	• Recognize early shock physiology. • Utilize POCUS. • Order appropriate labs. • Initiate diuresis or inotropes. • Escalate to ICU.	• Cardiology attending • Echocardiogram displaying acute heart failure with reduced ejection fraction

*ACS, acute coronary syndrome; *CABG,* coronary artery bypass graft; *STEMI,* ST-elevation myocardial infarction; *HFrEF,* heart failure with reduced ejection fraction; *POCUS,* point of care ultrasound; *EKG,* electrocardiogram; *IC,* interventional cardiology; *ICU,* intensive care unit; *CTS,* cardiothoracic surgery; *RVR,* rapid ventricular response; *TAVR,* transcatheter aortic valve replacement

1. Post-TAVR Bradycardia: Recognition loss of capture with the transvenous pacing trainer, troubleshooting transvenous pacing malfunction, escalation to electrophysiology or the primary operator, and contingency planning.
2. Post-CABG Tamponade: Differentiation between stable pericardial effusion and tamponade physiology, coordination with intensivists and surgeons, and ordering focused diagnostics (bedside cardiac echocardiogram), while exhibiting professionalism and interpersonal communication skills with a difficult attending.
3. Atrial Fibrillation with RVR: Hemodynamic assessment, diagnostic reasoning, and execution of appropriate pharmacologic or electrical cardioversion strategies.
4. STEMI/Chest Pain Activation: Prioritization among medical management, activation of the catheterization lab pathway, and communication with nursing staff and family members.
5. Acute Decompensated HFrEF: Identification of early cardiogenic shock, point-of-care ultrasound assessment, and initiation of evidence-based medical therapy.

### Learning objectives

Learning objectives were synthesized from contributions by the simulation team, senior cardiology attendings and the chief cardiology fellow to align with ACGME cardiology milestones in acute clinical decision-making, interpersonal skills, communication, and evidence-based medicine with systems-based practice. Core objectives included:

- Rapid recognition and management of common post-procedural and acute cardiology emergencies.
- Application of diagnostic reasoning and evidence-based management under time pressure.
- Prioritization and triage of simultaneous cardiovascular crises.
- Leadership and communication within interprofessional teams.
- Procedural troubleshooting (pacing, pericardiocentesis preparation) and hemodynamic interpretation in real time.

### Facilitation and debriefing

Faculty facilitators and simulation staff observed learner performance during each scenario and debriefing, focusing on communication, prioritization, escalation behavior, and procedural troubleshooting. These observations were documented informally by facilitators and used to guide individualized feedback and identify areas for curricular refinement. No standardized observational rubric, validated assessment tool, audio/video transcription, or formal qualitative analytic method was used.

Sessions were facilitated by academic cardiology faculty who were the content experts and supplemented by simulation staff who served as embedded observers and role players. Nurses from the cardiac step-down and ICU units participated to enhance authenticity. Each fellow participated in a structured, individualized debriefing following completion of all five simulation scenarios. Debriefings were guided by the Promoting Excellence and Reflective Learning in Simulation (PEARLS) framework and incorporated principles of *Debriefing with Good Judgment* to promote reflection on the Cardiovascular Disease Milestones described above.

Faculty facilitators and simulation staff observed learner performance during each scenario and debriefing, focusing on communication, prioritization, escalation behavior, and procedural troubleshooting. These observations were documented informally by facilitators and used to guide individualized feedback and identify areas for curricular refinement. No standardized observational rubric, validated assessment tool, audio/video transcription, or formal qualitative analytic method was used. Nursing feedback was not systematically collected or analyzed in this pilot evaluation.

### Assessment

Learners completed pre- and post-simulation self-assessment surveys rating their confidence across several domains using a 5-point Likert scale (1 = not confident; 5 = very confident). The survey included individual items grouped into domains of clinical decision-making, procedural skills and troubleshooting, interprofessional communication, leadership and professionalism, scenario-specific confidence, and global confidence. Domain-level scores were calculated by averaging the individual item scores within each domain. Because of the small sample size and exploratory nature of this pilot evaluation, results were summarized primarily at the domain level.

Faculty facilitators documented informal observational impressions during simulation sessions and debriefings, focusing on communication, prioritization, escalation behavior, and procedural troubleshooting. These observations were used to guide individualized feedback and identify potential curricular gaps rather than serve as formal qualitative outcome measures. No formal qualitative analysis methodology, transcription, coding process, standardized rubric, or validated observational instrument was used.

The survey questionnaire was developed specifically for this study and has not been previously published. An English-language version of the survey instrument is provided as a supplementary file.

### Statistical analysis

Given the exploratory nature of this pilot educational evaluation and the small sample size, analyses were primarily descriptive. Pre- and post-simulation confidence ratings were summarized using mean Likert-scale scores across domains. Because only five fellows participated, formal hypothesis testing was not emphasized, and findings are presented descriptively to illustrate trends in learner self-reported confidence before and after the intervention. Individual learner score trajectories were additionally visualized to demonstrate within-participant changes across domains.

### Ethics approval and consent to participate

This project was conducted as a single-center educational innovation embedded within the cardiology fellowship onboarding program. The Institutional Review Board at Naples Comprehensive Health reviewed the study protocol and determined that formal ethics approval and informed consent were not required due to the retrospective design and use of de-identified data. The study was conducted in accordance with the principles of the Declaration of Helsinki.

## Results

### Participant characteristics

All five incoming first-year cardiology fellows at the NCH Rooney Heart Institute participated in the simulation as part of the 2025 orientation program. Two fellows (40%) had prior simulation exposure during internal medicine residency training at the same training hospital (median duration, 2–3 years), though none had previously engaged in cardiology-specific or night-float–style simulations. The remaining three fellows had no exposure to structured simulation training. All participants completed the simulation and structured debriefing session.

### Pre- and post-simulation confidence

**Fig. 1:**
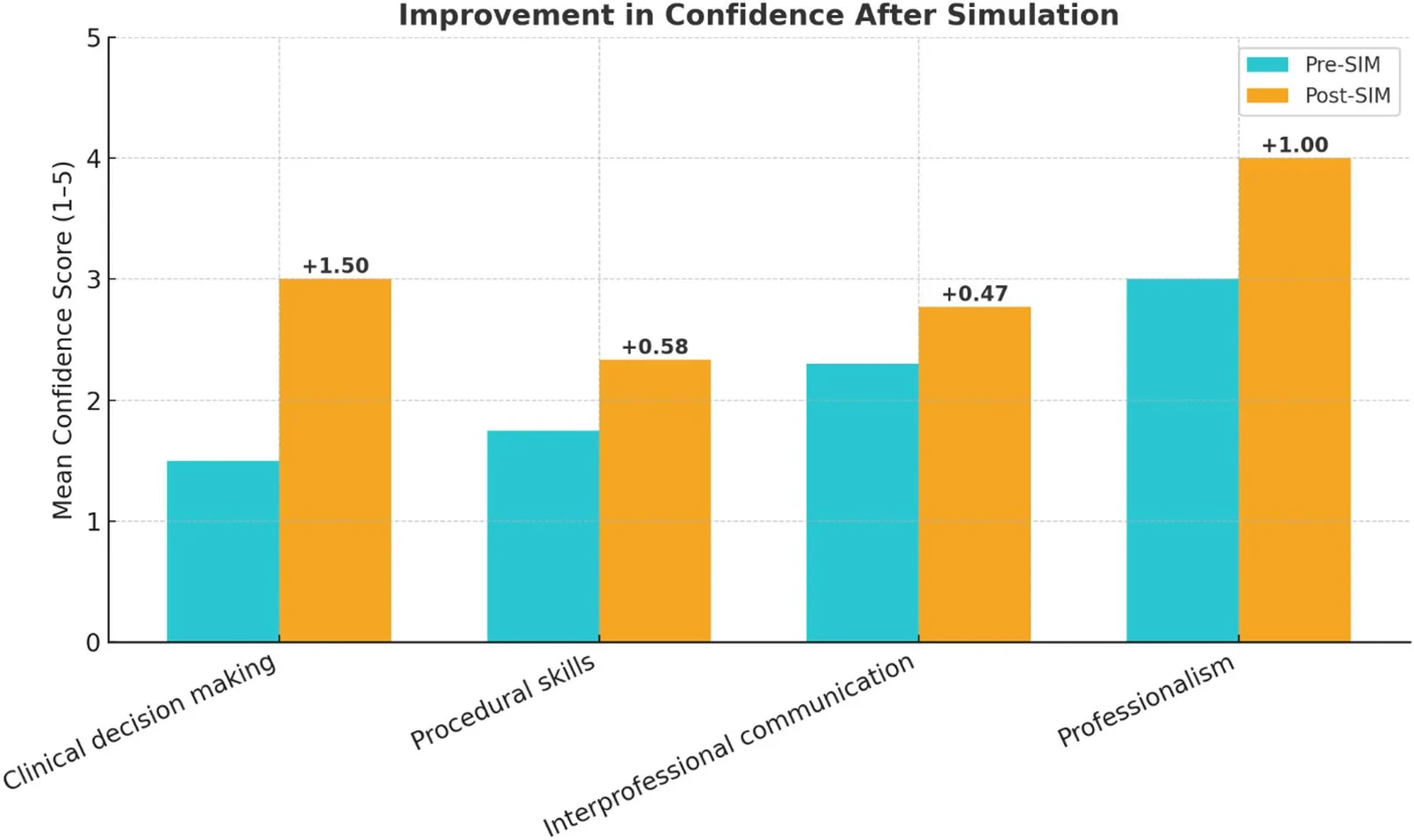
Improvement in Confidence After Simulation. Bar graph demonstrating mean Likert-scale confidence scores (1 = not at all confident, 2 = slightly confident, 3 = moderately confident, 4 = confident, 5 = very confident) across four domains before and after simulation-based training. Post-simulation scores (orange) increased in all areas compared with pre-simulation scores (teal), with the numeric labels above each bar pair denoting the mean improvement for that domain. These findings reflect measurable gains in self-perceived competence following the educational intervention

**Fig. 2:**
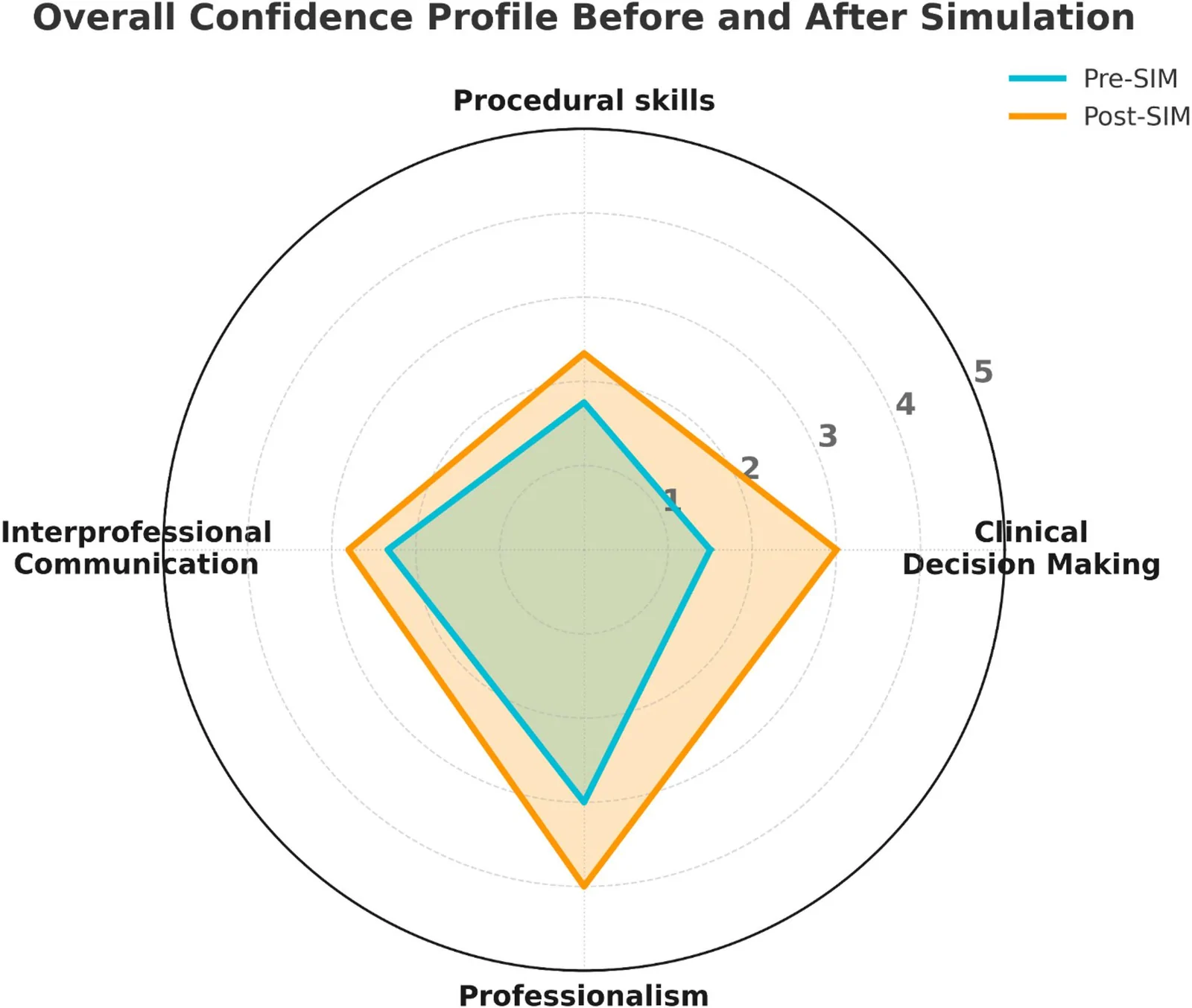
Overall Confidence Profile Before and After Simulation. This radar plot illustrates mean confidence ratings (Likert scale 1–5) across four core competency domains — Clinical Decision Making, Procedural Skills, Interprofessional Communication, and Professionalism — before and after participation in the simulation-based training. The orange area (Post-SIM) represents higher confidence scores across all domains compared with the teal area (Pre-SIM), indicating overall improvement in self-reported competency following the simulation exercise

**Fig. 3:**
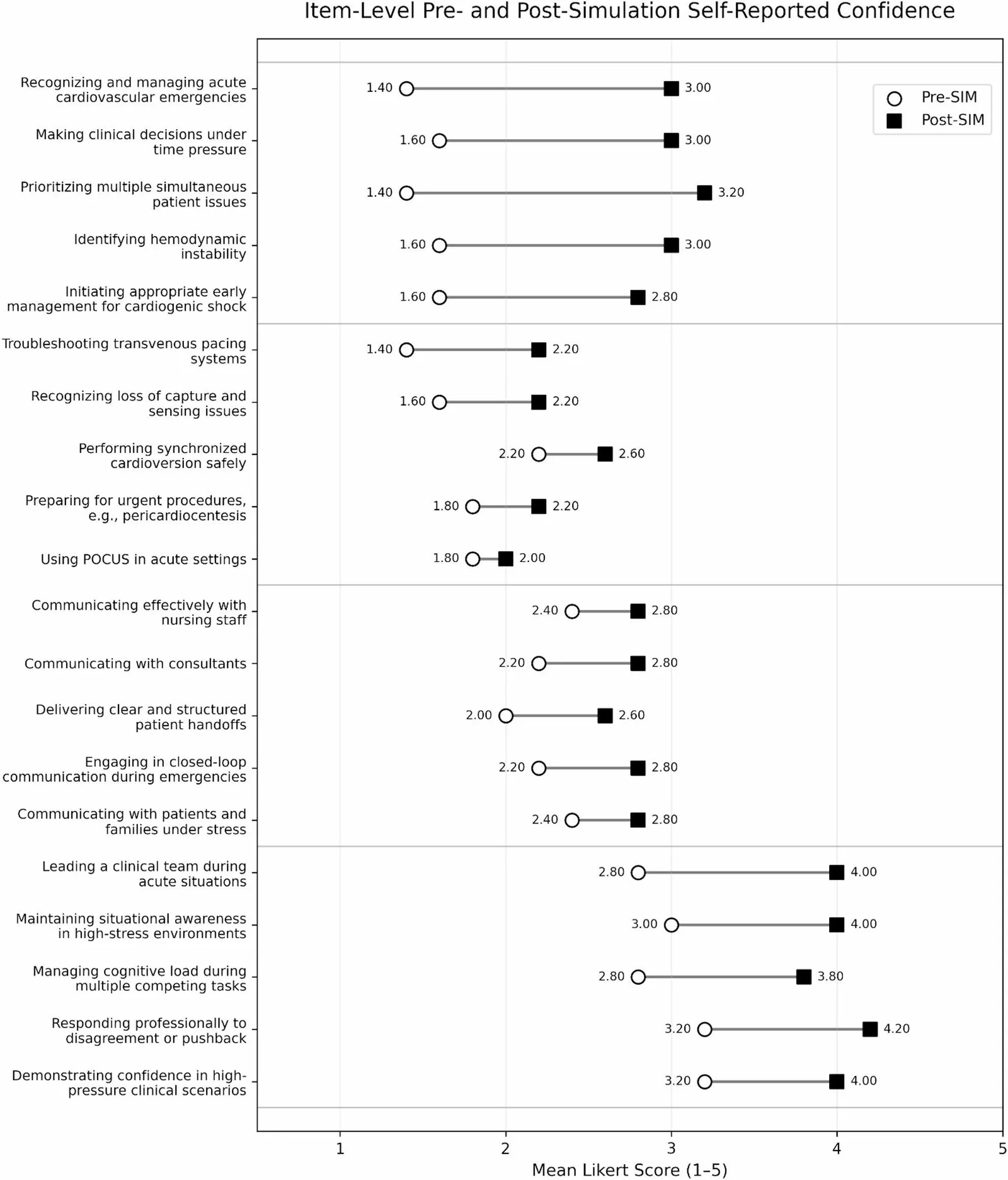
Item-Level Pre- and Post-Simulation Self-Reported Confidence Scores. Dumbbell plot displaying mean Likert-scale confidence scores for each individual survey item before and after participation in the cardiology night-float simulation. Open circles represent pre-simulation scores and filled squares represent post-simulation scores. Items are grouped by survey domain and separated by horizontal divider lines. Values reflect self-reported confidence among participating fellows and should be interpreted descriptively rather than as objective measures of skill acquisition or clinical performance

All five fellows demonstrated directional improvement in at least several confidence domains, although the magnitude of improvement varied between participants. (Figures 1 and 2). All confidence domains were assessed using a 5-point Likert scale (1 = not confident at all, 5 = extremely confident). Mean self-reported confidence in clinical decision-making increased from 1.5 to 3.0, representing the largest relative increase. Confidence in procedural skills improved from 1.75 to 2.25, while interprofessional communication increased from 2.25 to 2.75. Professionalism, which encompassed leadership, situational awareness, and communication under stress, increased from 3.0 to 4.0 (Figs. 1 and 2). Item-level pre- and post-simulation self-reported confidence scores are shown in Fig. 3. The largest descriptive increases were observed in prioritizing multiple simultaneous patient issues, recognizing and managing acute cardiovascular emergencies, making clinical decisions under time pressure, identifying hemodynamic instability, and leading a clinical team during acute situations.

Overall, fellows reported increased comfort managing complex overnight cardiology scenarios, particularly in areas related to pacing troubleshooting, triage prioritization, and closed-loop communication.

### Observed performance

Faculty observers noted strong engagement and progressive improvement in clinical performance as scenarios advanced, accompanied by enhanced situational awareness and the ability to manage cognitive load during complex, overlapping urgencies.

Fellows commonly encountered initial challenges troubleshooting the transvenous pacer box during the post-TAVR bradycardia scenario, with frequent difficulty confirming appropriate output settings and recognizing under- or oversensing. Despite these difficulties, each participant demonstrated appropriate escalation behavior and effectively communicated with any advanced consultants when encountering technical uncertainty.

During the tamponade scenario, fellows consistently responded to ICU attending pushback with professionalism and collaborative decision-making, effectively advocating for urgent evaluation while recognizing that pericardiocentesis was not indicated. In the STEMI activation case, three fellows—each trained at outside institutions—exhibited uncertainty in activating the catheterization laboratory and during sign-out to the interventional cardiologist.

Procedural performance improved across the session. Four fellows correctly prepared and delivered synchronized cardioversion for atrial fibrillation with rapid ventricular response, demonstrating strong safety awareness and effective closed-loop communication with nursing staff. In the final scenario, all fellows accurately identified cardiogenic shock, though only one initiated appropriate medical therapy with inotropic support during the initial management phase.

The involvement of academic faculty and the chief cardiology fellow in simulation facilitation allowed for direct observation of trainee performance in real time. Through this process, two fellows were identified for additional support and received follow-up by the senior cardiology fellows after their respective night-call experiences.

### Debriefing observations and learner reflections

During post-simulation debriefings, fellows frequently discussed several recurring areas of perceived educational value, including realism, prioritization under pressure, interdisciplinary communication, and procedural confidence. Several fellows also reported discomfort with transvenous pacemaker troubleshooting and uncertainty regarding escalation pathways during STEMI activation, particularly when adapting to unfamiliar institutional workflows. These reflections were collected informally during facilitator-led debriefings and were not analyzed using a formal qualitative methodology.

### Programmatic impact

Facilitators noted improvement in learners’ situational awareness, critical thinking, and leadership tone. The simulation also uncovered areas for curricular enhancement, including additional focus on transvenous pacing troubleshooting and early recognition of cardiogenic shock, which were subsequently incorporated into orientation didactics and skills labs.

## Discussion

In this preliminary educational evaluation, a high-fidelity, multi-scenario cardiology ‘night-float’ simulation was feasible to implement and was associated with improvements in fellows’ self-reported confidence across several domains, including clinical decision-making, procedural troubleshooting, communication, and professionalism. The structured sequence of acute cardiology cases mirrored the cognitive demands of an actual overnight call, providing a safe environment for deliberate practice and self-reflection [2, 3, 5, 14]. These findings should be interpreted as exploratory given the small cohort size, reliance on non-validated self-assessment measures, and absence of objective performance outcomes.

Consistent with prior literature, the largest relative increases in self-reported confidence were observed in clinical decision-making and leadership under pressure. Simulation-based training has been shown to increase learner confidence, improve crisis resource management, and strengthen diagnostic reasoning with the same results as traditional didactics [2, 4, 15]. Following the simulation, fellows reported increased confidence in triage prioritization, transvenous pacing troubleshooting, and interprofessional communication. These findings reflect self-perceived preparedness rather than objectively measured skill acquisition, as no validated performance assessment or repeat simulation session was used. These observations are broadly consistent with prior simulation-based education literature suggesting that exposure to acute cardiovascular scenarios may improve perceived readiness for call responsibilities [7, 13, 16, 17]. The first two weeks of overnight call represent one of the most anxiety-provoking and formative periods for new cardiology fellows, as they transition from supervised residency responsibilities to autonomous, high-stakes decision-making. During this time, fellows must manage multiple simultaneous emergencies, often with limited familiarity with institutional workflows or available support. In this context, simulation-based orientation may represent a useful adjunct to traditional fellowship onboarding and warrants further evaluation in larger, multi-center curricula. By allowing deliberate practice and exposure to realistic on-call scenarios within a safe environment, simulation-based orientation may help reduce early trainee anxiety and increase confidence before assuming overnight responsibilities, although effects on patient safety require further study.

The simulation was able to reveal specific gaps in individual fellow readiness for overnight call responsibilities, highlighting opportunities for focused and curricular refinement. All participants initially struggled with transvenous pacer troubleshooting, underscoring the need for deliberate instruction in transvenous pacing troubleshooting. Despite these technical challenges, all fellows demonstrated appropriate escalation behaviors and effective communication with the primary operator and electrophysiology team. During the tamponade scenario, fellows responded to ICU attending pushback with professionalism. This scenario provided an opportunity for facilitators to observe how trainees communicated clinical concerns, advocated for appropriate evaluation, and navigated disagreement in an interprofessional setting. By contrast, three fellows—each trained at outside institutions—hesitated to activate the catheterization laboratory and exhibited discomfort during handoff to the interventional cardiologist, reflecting interinstitutional variability in cardiac catheterization activation protocols and a need for early orientation standardization. In the final case, all fellows identified cardiogenic shock, though only one initiated timely inotropic therapy, suggesting a gap in pharmacologic decision-making under pressure.

These findings reinforce existing literature demonstrating that high-fidelity simulation not only improves learner confidence but also uncovers latent safety and performance gaps [3, 5, 12, 18]. The inclusion of standardized patients and real nursing staff enhanced psychological fidelity and realism, allowing fellows to experience both technical and interpersonal dimensions of clinical crises. Similar benefits of interprofessional simulation have been reported in other specialties, showing improved teamwork, communication, and mutual respect among healthcare team members [18].

The debriefing structure allowed learners to receive individualized feedback, reflect on decision-making, and discuss perceived strengths and areas for improvement. Integrating PEARLS and *Debriefing with Good Judgment* allowed facilitators to transform reflection into targeted learning, reinforcing clinical reasoning, team communication, and adaptive performance under cognitive load.

The strengths of this study include its integration into the fellowship orientation curriculum, individualized simulation design that minimized peer influence, and the use of both quantitative (Likert scale) and qualitative data (debriefing) to provide a comprehensive assessment of learning outcomes.

## Limitations

This study has several important limitations. It was a single-center educational innovation conducted at one academic institution with a small cohort of five incoming cardiology fellows, limiting generalizability to other training programs. The primary quantitative outcomes were self-reported confidence measures, which reflect perceived preparedness but not necessarily competence or sustained performance improvement. Objective metrics such as checklist-based scoring or timed task analysis were not used and should be considered for future work.

Another important limitation is that post-simulation debriefings were conducted in a non-anonymous setting and included cardiology faculty and fellowship leaders who held supervisory roles relative to the participating fellows. This may have introduced social desirability bias, as trainees may have been less likely to report negative perceptions, discomfort, or criticism of the simulation curriculum in the presence of individuals involved in their training or evaluation.

Additionally, the absence of longitudinal follow-up precludes assessment of knowledge retention or translation of simulation skills into real-world clinical performance.

Finally, this curriculum relied on locally available resources, including high-fidelity mannequins, standardized patients, and the transvenous pacing trainer. While these tools enhanced realism, their availability and fidelity may vary across institutions, potentially limiting reproducibility. Despite these limitations, the simulation effectively identified early performance gaps in pacing, defibrillation practice, and cardiogenic shock management—insights that can inform structured cardiology orientation curricula across programs.

## Conclusion

This pilot simulation curriculum suggests that a structured cardiology night-float orientation is feasible and may enhance learner confidence and identify early educational gaps among incoming fellows. Larger studies incorporating validated assessment tools and objective performance metrics are needed to determine educational impact and generalizability.

## Supplementary Information

Supplementary Material 1.

## Data Availability

The datasets generated and/or analyzed during the current study are not publicly available due to the small sample size and potential risk of participant identification but are available from the corresponding author on reasonable request in de-identified form.
