# Supplementary material for: A fellows first night on call: managing the chaos in the storm
Source: BMC Med Educ. 2026 Jun 30;26:1417. doi: 10.1186/s12909-026-09623-8 (PMC13540915; doi:10.1186/s12909-026-09623-8)
Supplement: Supplementary file 1 [file 12909_2026_9623_MOESM1_ESM.docx]

**Cardiology Night-Float Simulation Survey**

***Sections 1-4 were conducted pre and post simulation.***

**Instructions**

Please rate your confidence in each of the following domains.

Use the scale:
1 = Not confident at all
2 = Slightly confident
3 = Moderately confident
4 = Confident
5 = Very confident

**Section 1: Clinical Decision-Making**

1. Recognizing and managing acute cardiovascular emergencies
2. Making clinical decisions under time pressure
3. Prioritizing multiple simultaneous patient issues
4. Identifying hemodynamic instability
5. Initiating appropriate early management for cardiogenic shock

**Section 2: Procedural Skills & Troubleshooting**

6. Troubleshooting transvenous pacing systems
7. Recognizing loss of capture and sensing issues
8. Performing synchronized cardioversion safely
9. Preparing for urgent procedures (e.g., pericardiocentesis)
10. Using point-of-care ultrasound (POCUS) in acute settings

**Section 3: Interprofessional Communication**

11. Communicating effectively with nursing staff
12. Communicating with consultants (e.g., EP, interventional cardiology)
13. Delivering clear and structured patient handoffs
14. Engaging in closed-loop communication during emergencies
15. Communicating effectively with patients and families under stress

**Section 4: Leadership & Professionalism**

16. Leading a clinical team during acute situations
17. Maintaining situational awareness in high-stress environments
18. Managing cognitive load during multiple competing tasks
19. Responding professionally to disagreement or pushback
20. Demonstrating confidence in high-pressure clinical scenarios

**Section 5: Scenario-Specific Confidence**

21. Managing post-TAVR bradycardia and pacing complications
22. Evaluating and managing suspected cardiac tamponade
23. Managing atrial fibrillation with rapid ventricular response
24. Activating the cath lab for suspected STEMI
25. Managing acute decompensated heart failure

**Section 6: Global Confidence**

26. Overall confidence managing overnight cardiology call
27. Readiness to independently triage acute cardiology patients
28. Comfort managing multiple simultaneous emergencies

**Section 7: Qualitative Feedback (Post-Simulation Only)**

**Free Response:**

1. What aspects of the simulation were most helpful?
2. What aspects were most challenging?
3. What skills do you feel improved the most?
4. What areas do you still feel least confident in?
5. Suggestions for improving the simulation experience

| Domain | Survey item | Pre-SIM mean | Post-SIM mean | Mean change |
| --- | --- | --- | --- | --- |
| Clinical decision-making | Recognizing and managing acute cardiovascular emergencies | 1.40 | 3.00 | +1.60 |
| Clinical decision-making | Making clinical decisions under time pressure | 1.60 | 3.00 | +1.40 |
| Clinical decision-making | Prioritizing multiple simultaneous patient issues | 1.40 | 3.20 | +1.80 |
| Clinical decision-making | Identifying hemodynamic instability | 1.60 | 3.00 | +1.40 |
| Clinical decision-making | Initiating appropriate early management for cardiogenic shock | 1.60 | 2.80 | +1.20 |
| Clinical decision-making mean |  | **1.52** | **3.00** | **+1.48** |
| Procedural skills/troubleshooting | Troubleshooting transvenous pacing systems | 1.40 | 2.20 | +0.80 |
| Procedural skills/troubleshooting | Recognizing loss of capture and sensing issues | 1.60 | 2.20 | +0.60 |
| Procedural skills/troubleshooting | Performing synchronized cardioversion safely | 2.20 | 2.60 | +0.40 |
| Procedural skills/troubleshooting | Preparing for urgent procedures, e.g., pericardiocentesis | 1.80 | 2.20 | +0.40 |
| Procedural skills/troubleshooting | Using POCUS in acute settings | 1.80 | 2.00 | +0.20 |
| Procedural skills/troubleshooting mean |  | **1.76** | **2.24** | **+0.48** |
| Interprofessional communication | Communicating effectively with nursing staff | 2.40 | 2.80 | +0.40 |
| Interprofessional communication | Communicating with consultants | 2.20 | 2.80 | +0.60 |
| Interprofessional communication | Delivering clear and structured patient handoffs | 2.00 | 2.60 | +0.60 |
| Interprofessional communication | Engaging in closed-loop communication during emergencies | 2.20 | 2.80 | +0.60 |
| Interprofessional communication | Communicating with patients and families under stress | 2.40 | 2.80 | +0.40 |
| Interprofessional communication mean |  | **2.24** | **2.76** | **+0.52** |
| Leadership/professionalism | Leading a clinical team during acute situations | 2.80 | 4.00 | +1.20 |
| Leadership/professionalism | Maintaining situational awareness in high-stress environments | 3.00 | 4.00 | +1.00 |
| Leadership/professionalism | Managing cognitive load during multiple competing tasks | 2.80 | 3.80 | +1.00 |
| Leadership/professionalism | Responding professionally to disagreement or pushback | 3.20 | 4.20 | +1.00 |
| Leadership/professionalism | Demonstrating confidence in high-pressure clinical scenarios | 3.20 | 4.00 | +0.80 |
| Leadership/professionalism mean |  | **3.00** | **4.00** | **+1.00** |

**Supplementary Table 2. Item-Level Pre- and Post-Simulation Self-Reported Confidence Scores.** Individual survey items are grouped by domain. Values represent mean Likert-scale scores among participating fellows before and after simulation, with mean change calculated as post-simulation minus pre-simulation score.
